# Effective fitness under fluctuating selection with genetic drift

**DOI:** 10.1093/g3journal/jkad230

**Published:** 2023-10-10

**Authors:** Siliang Song, Jianzhi Zhang

**Affiliations:** Department of Ecology and Evolutionary Biology, University of Michigan, Ann Arbor, MI 48109, USA; Department of Ecology and Evolutionary Biology, University of Michigan, Ann Arbor, MI 48109, USA

**Keywords:** natural selection, fluctuating environment, genetic drift, geometric mean fitness, effective fitness

## Abstract

The natural environment fluctuates for virtually every population of organisms. As a result, the fitness of a mutant may vary temporally. While commonly used for summarizing the effect of fluctuating selection on the mutant, geometric mean fitness can be misleading under some circumstances due to the influence of genetic drift. Here, we show by mathematical proof and computer simulation that, with genetic drift, the geometric mean fitness does not accurately reflect the overall effect of fluctuating selection. We propose an alternative measure based on the average expected allele frequency change caused by selection and demonstrate that this measure—effective fitness—better captures the overall effect of fluctuating selection in the presence of drift.

## Introduction

**Fig. 1.**
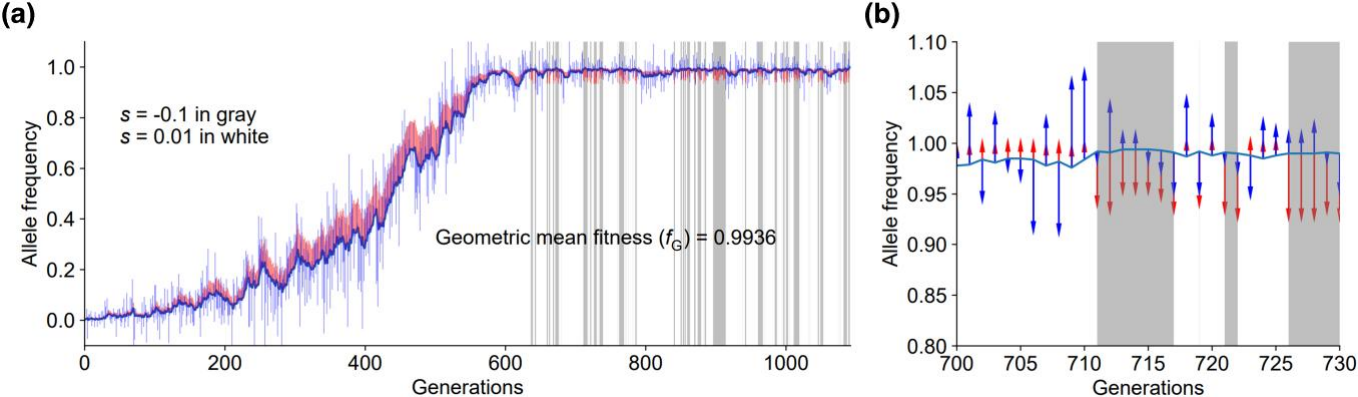
An example where the geometric mean fitness (*f*_G_) of the mutant allele is below 1, yet the mutant frequency changes suggest positive selection. Blue and red vertical bars represent allele frequency changes per generation caused by drift (×10) and selection (×50), respectively, with each bar pointing to the direction of the allele frequency change in the corresponding generation. a) The full allele frequency trajectory of a newly arisen allele that eventually becomes fixed. b) The allele frequency trajectory from the 700th to 730th generation is enlarged to show the effects of selection (red arrows) and drift (blue arrows). The simulation is carried out with a haploid Wright–Fisher population of *N*_e_ = 1,000. A frequency-dependent selection is simulated where the mutant allele has a relative fitness of 1.01 (or selection coefficient *s* = 0.01) when the allele frequency is below 0.99 (indicated by white regions); otherwise, the allele has a relative fitness of 0.9 (indicated by gray regions).

Very few natural populations can be said to live in a constant environment. Environmental changes are virtually ubiquitous and come in many forms, including both relatively rapid changes that occur many times in the life of an organism and relatively slow changes that are difficult to sense in one generation. Because the fitness of a mutant is often environment-dependent, natural selection acting on a mutant is expected to fluctuate with time as the environment changes (Bell 2010; Rudman et al. 2022). Fluctuating selection has been studied both theoretically (Gillespie 1973; Karlin and Levikson 1974; Gillespie 1991; Bürger and Lynch 1995; Cvijović et al. 2015) and empirically (Cooper and Lenski 2010; Lambert and Kussell 2014; Abdul-Rahman et al. 2021; Machado et al. 2021; Rudman et al. 2022). Despite the temporal variation of a mutant's fitness, it is useful to have a single value that captures the overall selection acting on the mutant over multiple generations. Geometric mean fitness over generations (*f*_G_) is widely used for this purpose. However, in a population genetic simulation, we found *f*_G_ misleading in some cases. For example, Fig. 1 shows a case of a newly arisen mutant that was eventually fixed in a haploid population with an effective population size (*N*_e_) of 10^3^. The mutant was under fluctuating selection with a selection coefficient (*s*) of either 0.01 (white regions in Fig. 1) or −0.1 (gray regions). We computed that *f*_G_ of the mutant, relative to the wild type, was 0.9936 from its appearance to fixation. However, plotting the mutant frequency over time revealed a rapid frequency increase in a short time, apparently driven by positive selection (Fig. 1). But why was its *f*_G_ below 1? It turns out that, in this case, negative selection acted on the mutant when its frequency was very close to 1, while positive selection acted when its frequency was intermediate. It is known that when the mutant frequency is close to 0 $\left(<1/(N_{e}|s|)\right)$ or 1 $\left(>1-1/(N_{e}|s|)\right)$, selection is overshadowed by genetic drift (Desai and Fisher 2007). Hence, negative selection had a limited effect on the mutant frequency in this example. Because *f*_G_ is computed without the consideration of the mutant frequency, *f*_G_ may not accurately reflect the overall selective effect.

The above example raises the question of whether mutant frequencies should be considered in calculating a mutant's overall fitness in a fluctuating environment. In this study, we demonstrate the limitation of *f*_G_ in capturing the overall effect of fluctuating selection in the presence of genetic drift and propose an alternative measure—effective fitness—based on the average expected mutant frequency change due to selection. Throughout this study, fitness refers to expected fitness rather than realized fitness.

## Materials and methods

### Expected mutant frequency under fluctuating selection with genetic drift does not equal that without drift

Let us consider 2 generations of evolution for a haploid population with an effective population size of *N*_e_. A mutant has an initial frequency of $q_{0}$ and a fitness (relative to the wild type) of $f_{1}$ and $f_{2}$ in the first and second generation, respectively.

Under the standard Wright–Fisher model, the number of mutant copies after 1 generation of selection (*m*_1_) follows the binomial distribution given by $B(N_{e},(q_{0}f_{1}/(1+q_{0}(f_{1}-1))))$. Therefore, the mutant frequency after the first generation, $q_{1}=m_{1}/N_{e}$, is a random variable following a scaled binomial distribution with the mean of $E(q_{1})=q_{0}f_{1}/(1+q_{0}(f_{1}-1))$.

Given *q*_1_, the number of mutants after another generation of selection (*m*_2_) follows the binomial distribution $B(N_{e},(q_{1}f_{2}/(1+q_{1}(f_{2}-1))))$. Therefore, the expected mutant frequency after the second generation is


$$\begin{array}{c} E(q_{2})=\frac{E(m_{2})}{N_{e}}=E\left(\frac{q_{1}f_{2}}{1+q_{1}(f_{2}-1)}\right) \end{array}$$ (1)

The function $F(x)=(xf_{2}/(1+x(f_{2}-1)))$ is concave when $f_{2}>1$ and convex when $f_{2}<1$, for x∈[0,1]. According to Jensen's inequality (Jensen 1906), when F(x) is concave, E(F(x))≤F(E(x)). The equality in the above formula will not hold in the present case, because F(x) is not linear in any region of x∈[0,1] and *x* = $q_{1}$ is a random variable potentially ranging from 0 to 1. Therefore, when F(x) is concave (when $f_{2}>1$), we have


$$\begin{array}{c} E(F(q_{1}))<F(E(q_{1})) \end{array}$$ (2)

On the left of (2), we have


$$E(F(q_{1}))=E\left(\frac{q_{1}f_{2}}{1+q_{1}(f_{2}-1)}\right)=\;E(q_{2})$$

On the right of (2), we have


$$\begin{array}{rl} F(E(q_{1})) & =F\left(\frac{q_{0}f_{1}}{1+q_{0}(f_{1}-1)}\right)\\ & =\frac{\frac{q_{0}f_{1}}{1+q_{0}(f_{1}-1)}f_{2}}{1+\frac{q_{0}f_{1}(f_{2}-1)}{1+q_{0}(f_{1}-1)}}\\ & =\frac{q_{0}f_{1}f_{2}}{1+q_{0}(f_{1}-1)+q_{0}f_{1}(f_{2}-1)}\\ & =\frac{q_{0}f_{1}f_{2}}{1+q_{0}(f_{1}f_{2}-1)} \end{array}$$

Therefore, (2) becomes


$$\begin{array}{c} E(q_{2})<\frac{q_{0}f_{1}f_{2}}{1+q_{0}(f_{1}f_{2}-1)} \end{array}$$ (3)

Similarly, when F(x) is convex (when $f_{2}<1$), we have


$$\begin{array}{c} E(q_{2})>\frac{q_{0}f_{1}f_{2}}{1+q_{0}(f_{1}f_{2}-1)} \end{array}\;$$ (4)

Now let us derive $q_{2}^{\prime }$, the analytical expression of the mutant frequency after the second generation without genetic drift. Under the same initial mutant frequency and 2 generations of selection, it is easy to show that $q_{2}^{\prime }=(q_{0}f_{1}f_{2}/(1+q_{0}(f_{1}f_{2}-1)))$.

Note that $q_{2}^{\prime }$ equals the right side of (3) and (4). So, (3) and (4) now become


$$\{\begin{array}{cc} E(q_{2})<q_{2}^{\prime } & \mathrm{if}f_{2}>1\\ E(q_{2})>q_{2}^{\prime } & \mathrm{if}f_{2}<1 \end{array}$$ (5)

Hence, the expected mutant frequency under 2-generation fluctuating selection with drift does not equal that without drift.

### Expected mutant frequency under fluctuating selection does not equal that under constant selection with *f*_G_ when there is drift

We now evaluate whether the expected mutant frequency after fluctuating selection with drift equals that after constant selection with *f*_G_ in the presence of drift. Again, for simplicity, we consider 2 generations. We note that *f*_1_ and *f*_2_ can be rewritten as


$$\{\begin{array}{c} f_{1}=e^{x}f_{G}\\ f_{2}=e^{-x}f_{G} \end{array}$$

where x∈R. We rewrite the formula in this way so that $f_{1}$ and $f_{2}$ can be expressed using $f_{G}$ and a variable *x*, where *x* controls the level of difference between $f_{1}$ and $f_{2}$. When x>0, $f_{1}>f_{G}>f_{2}$; when x<0, $f_{1}<f_{G}<f_{2}$; and when x=0, $f_{1}=f_{2}=f_{G}$. With the new notation, Eq. (1) becomes $E(q_{2})=E(\left(q_{1}e^{-x}f_{G})/(1+q_{1}(e^{-x}f_{G}-1)\right))$, where $q_{1}=m_{1}/N_{e}$ and $m_{1}∼B(N_{e},(q_{0}e^{x}f_{G}/(1+q_{0}(e^{x}f_{G}-1))))$. We can view $E(q_{2})$ in the above as a function *G* of *x*. That is,


$$\begin{array}{rl} G(x) & =E\left(\frac{q_{1}e^{-x}f_{G}}{1+q_{1}(e^{-x}f_{G}-1)}\right)\\ & =\overset{N_{e}}{\underset{i=0}{\sum }}\;[\left(\begin{array}{c} N_{e}\\ i \end{array}\right){\left(\frac{q_{0}e^{x}f_{G}}{1+q_{0}(e^{x}f_{G}-1)}\right)}^{i}{\left(\frac{1-q_{0}}{1+q_{0}(e^{x}f_{G}-1)}\right)}^{N_{e}-i}\\ & \;\left(\frac{\frac{i}{N_{e}}e^{-x}f_{G}}{1+\frac{i}{N_{e}}(e^{-x}f_{G}-1)}\right)] \end{array}$$

We now hope to prove that G(x)≠G(0) for any x≠0 across the parameter space ($N_{e}$, $q_{0}$, $f_{G}$). Most straightforwardly, if we can prove that G(x) is a strictly monotonic function of *x*, the problem is solved. However, we found it difficult to analytically prove the monotonicity of G(x). Instead, we attempted to demonstrate the monotonicity of G(x) numerically. To explore various combinations of parameters $q_{0}$, $f_{G}$, and $N_{e}$, we varied each parameter individually while holding the other 2 parameters constant. Specifically, $q_{0}$ varied from 0.1 to 0.9 with an interval of 0.1, while $f_{G}$ and $N_{e}$ were fixed at 0.9 and 1,000, respectively (Supplementary Fig. 1a); log_2_  $f_{G}$ varied from −1 to 1 with an interval of 0.2, while $q_{0}$ and $N_{e}$ were fixed at 0.3 and 1,000, respectively (Supplementary Fig. 1b); $N_{e}$ varied from 1,000 to 10,000 with an interval of 1,000, while $q_{0}$ and $f_{G}$ were fixed at 0.3 and 0.9, respectively (Supplementary Fig. 1c). The G(x) curve was drawn by sampling *x* from −1 to 1 with an interval of 0.02. The monotonicity of G(x) was then inspected visually.

### Population genetic simulations with *f*_G_ = 1 or *f*_E_ = 1

We conducted population genetic simulations to evaluate the accuracy of using *f*_G_ and effective fitness *f*_E_ (see *Results*) to infer allele frequency changes. In a haploid population of *N*_e_ = 1,000, we simulated a mutant with an initial frequency of 0.5 subject to 2*n* generations of fluctuating selection. To control *f*_G_ at exactly 1, we assigned the mutant a fitness of $f_{1}$ = 5/6 for the first *n* generations and $f_{2}$ = 6/5 for the next *n* generations. In an alternative scenario, the relative fitness of the mutant was 5/6 in generations 1, 3, 5, etc. and 6/5 in generations 2, 4, 6, etc. for a total of 2*n* generations.

To further validate that the bias in allele frequency inference from *f*_G_ is due to the nonlinearity between the current mutant frequency (*q*) and the allele frequency change caused by selection ($\Delta_{s}$), we specified a hypothetical linear relationship between *q* and $\Delta_{s}$ in the simulation: $\Delta_{s}=(f-1)q$, or $E(q_{1})=fq_{0}$, where $q_{0}$ is the current mutant frequency and $E(q_{1})$ is the expected mutant frequency in the next generation.

To control *f*_E_ at exactly 1, we assigned the mutant with the fitness that leads to an expected mutant frequency change caused by selection to be $\Delta_{s}$ = −0.045 in each generation for *n* generations, and then the fitness that leads to $\Delta_{s}$ = 0.045 in each generation for another *n* generations. We chose $\Delta_{s}$ in each of the first *n* generations to be −0.045 because the corresponding fitness that generates such an allele frequency change at an initial allele frequency of 0.5 is $((0.5+\Delta_{s})/(0.5-\Delta_{s}))\approx 5/6$, which is close to *f*_1_, making the 2 simulations comparable. Similarly, the corresponding fitness that generates $\Delta_{s}$ = 0.045 at an initial allele frequency of 0.5 is close to *f*_2_. We varied *n* from 1 to 7. For each *n*, we conducted 500,000 simulation runs to obtain the mean and 95% confidence interval. We also reported statistical significance using the *t*-test evaluating whether the observed mutant frequency after the fluctuating selection deviates significantly from 0.5. We discarded simulation runs in which the mutant was fixed or lost before the end of 2*n* generations because *f*_G_ or *f*_E_ did not equal 1 over the generations considered in these runs. These discarded runs included 0 runs in the simulation of $f_{G}=1\;$ and 18 runs in the simulation of $f_{E}\;$ = 1.

### Population genetic simulations under fluctuating selection

We conducted simulations to assess the consistency of $f_{E}$ or $f_{G}$ with the mutant frequency changes under fluctuating selection with drift. In a haploid population of *N*_e_ = 1,000, we started the simulation with an allele frequency of 0.01 or 0.5. At each generation, there was a probability of 0.05 for the relative fitness of the allele to switch between $f_{1}=11/10$ and $f_{2}=10/11$ (such that $f_{1}f_{2}=1$). The initial fitness of the allele was randomly assigned to be either $f_{1}$ or $f_{2}$, and the population evolved for 200 generations. We considered the first 500 simulation runs with the final mutant frequency of exactly 0.100 and calculated the expected mutant frequency (in the absence of drift) inferred by *f*_E_ or *f*_G_ in each of these runs. Specifically, given *f*_E_, the expected mutant frequency can be calculated by $q_{E}=q_{0}+200\bar{\Delta_{s}}$, where $q_{0}=0.01$, and $\bar{\Delta_{s}}=((f_{E}-1)/(2(f_{E}+1)))$; given *f*_G_, the expected mutant frequency can be calculated by $q_{G}=(q_{0}f_{G}^{200}/(1+q_{0}(f_{G}^{200}-1)))$, where $q_{0}=0.01$. We similarly examined simulation runs that resulted in the final mutant frequency of exactly 0.200, 0.300, …, or 0.900.

Additionally, we assessed the performance of $f_{E}$ and $f_{G}$ in inferring the evolutionary outcome of a mutant (fixed or lost) under fluctuating selection with drift. The same simulations were carried out with an initial mutant frequency of 0.01 or 0.5. When the allele became fixed or lost, we stopped the simulation and recorded $f_{E}$, $f_{G}$, and the evolutionary outcome (fixed/lost). We collected 5,000 simulation runs that resulted in mutant allele fixation and loss, respectively, from which the area under the receiver operating characteristic (ROC) curve, or AUC, was calculated for $f_{E}$ and $f_{G}$. Because the difference in AUC between $f_{E}$ and $f_{G}$ is small when the initial mutant frequency is 0.5, we further focused on the simulation runs where $f_{E}$ contradicts $f_{G}$. We collected 5,000 simulation runs that resulted in $f_{E}>1$ and $f_{G}<1$ and another 5,000 simulation runs that resulted in $f_{E}<1$ and $f_{G}>1$ to examine which category showed more fixations than losses.

### Population genetic simulation of newly arisen alleles under fluctuating selection

All simulation details were the same as described in the preceding section, except that we introduced a single copy of an allele into the population, instead of setting the allele frequency at 0.01 or 0.5 to start the simulation. When the allele became fixed or lost, we stopped the simulation and recorded $f_{E}$, $f_{G}$, *C*, and the evolutionary outcome (fixed/lost), where *C* is the relative selection effect (see *Results*). We conducted simulations and collected the relevant data from 5,000 runs that resulted in the fixation of the allele and 5,000 runs that resulted in the loss of the allele.

## Results

### Geometric mean fitness is a biased measure of selection in the presence of drift

**Fig. 2.**
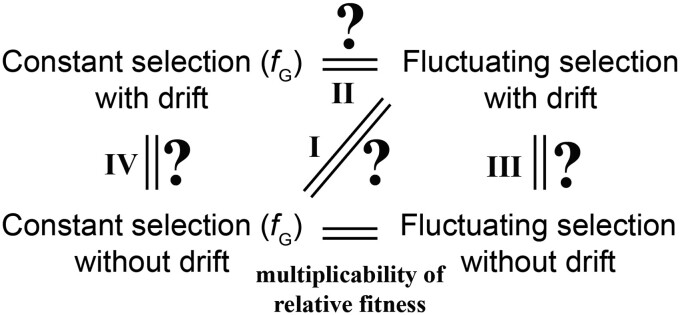
Relationships among different selection scenarios (constant vs. fluctuating selection) with and without genetic drift. The equal sign at the bottom of the figure represents the well-known conclusion that the allele frequency change under fluctuating selection without drift equals that under constant selection with the geometric mean fitness (*f*_G_) without drift, or the multiplicability of relative fitness. We examine in this study whether the expected allele frequency change under fluctuating selection with drift equals that under constant selection with *f*_G_, either with (indicated by question II) or without (indicated by question I) genetic drift. Because of the multiplicability of relative fitness, answering question I is equivalent to answering question III.

In a haploid population, in generation *i*, let the frequency of a mutant be *q_i_* and the relative fitness of the mutant be $f_{i}$. In the absence of genetic drift (e.g. when *N*_e_ approaches infinity), the initial mutant frequency *q*_0_ and the mutant frequency after *n* generations of selection satisfy the equation *q_n_*/(1 − *q_n_*) = [*q*_0_/(1 − *q*_0_)] $\prod_{i=1}^{n}f_{i}$ = [*q*_0_/(1 − *q*_0_)] $f_{G}^{n}$, where $f_{G}={\left({\prod_{i=1}^{n}f_{i}}_{i}\right)}^{1/n}$ is the geometric mean of the mutant fitness across *n* generations. This is why, in the absence of drift, fluctuating selection is equivalent to a constant selection with the mutant fitness equal to *f*_G_, as indicated by the equal sign at the bottom of Fig. 2.

However, when genetic drift occurs, it is unclear whether the expected mutant frequency change under fluctuating selection equals that caused by a constant selection with *f*_G_ in the presence (denoted by question II in Fig. 2) or absence (question I in Fig. 2) of genetic drift. As is clear from Fig. 2, by transitivity, evaluating question I is equivalent to evaluating whether the expected mutant frequency change under fluctuating selection with genetic drift equals that without genetic drift (question III in Fig. 2). Below, we demonstrate that the answer to question III is “no” and, thereby, the answer to question I is also “no”.

Let us consider 2 generations of evolution for a haploid population with an effective population size of *N*_e_. A mutant has an initial frequency of $q_{0}$ and a fitness (relative to the wild type) of $f_{1}$ and $f_{2}$ in the first and second generation, respectively. The mutant frequency becomes *q*_1_ and *q*_2_ after 1 and 2 generations, respectively. First, we derived $E(q_{2})$, the expected mutant frequency after the second generation in the presence of genetic drift (see *Materials and Methods*). Second, we derived the analytical expression of the mutant frequency after the second generation in the absence of genetic drift, $q_{2}^{\prime }$. Lastly, we found that $E(q_{2})$ does not equal $q_{2}^{\prime }$ when *f*_2_ ≠ 1. Specifically,


$$\{\begin{array}{cc} E(q_{2})<q_{2}^{\prime } & \mathrm{if}f_{2}>1\\ E(q_{2})>q_{2}^{\prime } & \mathrm{if}f_{2}<1 \end{array}$$

Interestingly, whether $E(q_{2})$ is greater or smaller than $q_{2}^{\prime }$ depends on $f_{2}\;$ but not $f_{1}$. This is because, in the presence of drift, selection still results in an unbiased expected $\;q_{1}$ (i.e. the expected $\;q_{1}\;$ with drift equals that without drift) when $q_{0}$ is given. Yet, because $\;q_{1}$ is a random variable, selection in the presence of drift results in a biased expected $q_{2}\;$ (i.e. the expected *q*_2_ does not equal $q_{2}^{\prime }$). Specifically, drift is expected to dampen the effect of selection (in the second generation). Another observation is that the above bias stems only from the nonlinear component of the relationship between the current allele frequency (*q*) and the effect of selection on the allele frequency ($\Delta_{s}=(q(1-q)(f-1))/(1+q(f-1))$, the subscript “s” indicates selection; see *Materials and Methods*), with no contribution from the linear component. To put it differently, if we could devise an evolutionary rule where $\Delta_{s}$ varies linearly rather than nonlinearly with *q*, the bias would disappear. We have numerically validated this point later in this section.

Therefore, the answer to question III is “no”, and by transitivity, the answer to question I is also “no”. Because constant selection with drift (top left in Fig. 2) is a special case of fluctuating selection with drift (top right in Fig. 2) and because the answer to question III is “no”, the expected mutant frequency after 2 generations of constant selection with drift does not equal that without drift (i.e. “no” to question IV in Fig. 2).

We next evaluated whether the expected mutant frequency after fluctuating selection with drift for 2 generations equals that after constant selection with *f*_G_ in the presence of drift (II in Fig. 2). This, however, was difficult to accomplish analytically. Instead, we evaluated it numerically (see *Materials and Methods* and Supplementary Fig. 1 for detailed analysis) and found that, in the presence of drift, the expected mutant frequency after 2 generations of fluctuating selection does not equal that after 2 generations of constant selection with *f*_G_. In other words, the answer to question II is also “no”.

**Fig. 3.**
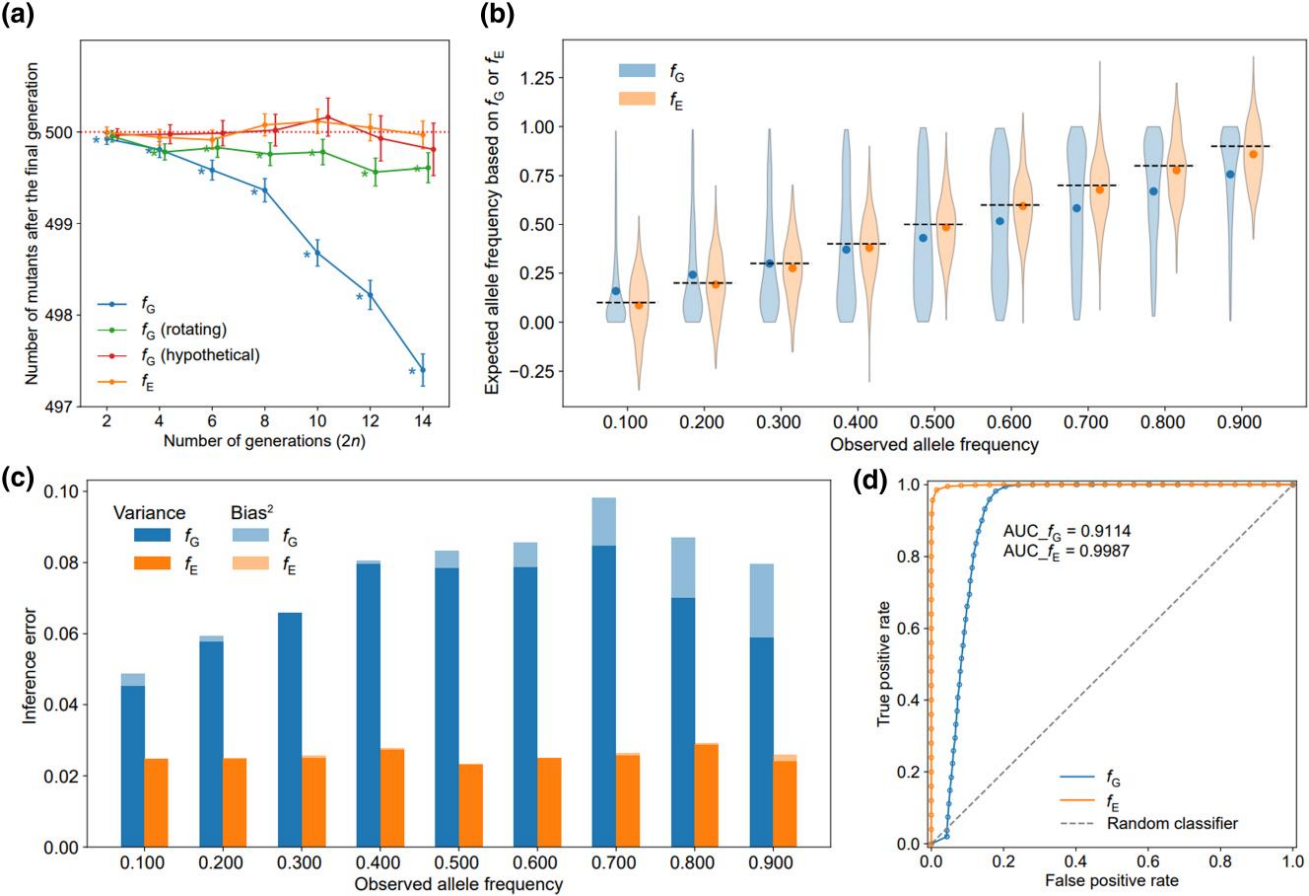
Performance of effective fitness (*f*_E_) and geometric mean fitness (*f*_G_) in inferring the outcome of simulated evolution. a) The mean number of mutants in 500,000 simulations of *n* generations of negative selection followed by *n* generations of positive selection in the presence of drift, with *f*_E_ = 1 (orange line) or *f*_G_ = 1 (blue line). We also simulated a scenario where negative selection acted in generations 1, 3, 5, etc. while positive selection acted in generations 2, 4, 6, etc. (green line). To show that the bias in *f*_G_ is caused by the nonlinear relationship between the mutant frequency change due to selection ($\Delta_{s}$) and the current mutant frequency (*q*), simulations of *f*_G_ = 1 were also conducted under the hypothetical scenario (red line) that $\Delta_{s}$ is a linear function of *q* ($\Delta_{s}=(f-1)q$). The mean number of mutants after 2*n* generations is lower than the initial value (horizontal dotted line) when *f*_G_ = 1 (blue and green lines). By contrast, the mean number is close to the initial value when *f*_E_ = 1 (orange line). The error bar represents the 95% confidence interval of the mean, whereas the asterisk indicates that the mean is statistically different from the initial value. b) Distribution of the expected mutant frequency inferred by *f*_G_ (blue) or *f*_E_ (orange) among simulation runs that resulted in a given final mutant frequency. Dots represent distribution means, whereas the horizontal dashed lines indicate observed final mutant frequencies. c) Errors of mutant frequency inference. The variance and bias^2^ of the estimation are shown using stacked bars, where the total stacked height represents the mean squared error (MSE). See Supplementary Fig. 3 for a clearer distribution of bias^2^. d) ROC curves and the corresponding AUC values show a stronger discriminative power of *f*_E_ than *f*_G_ in inferring the evolutionary outcomes of 100,000 simulation runs with an initial mutant frequency of 0.01.

Although we studied for only 2 generations when addressing questions I to IV, it is clear that the answers to these questions will remain “no” when additional generations are considered. It is likely that, under fluctuating selection with drift, *f*_G_-based inference of mutant frequency will deviate more from its true value as the number of generations rises. To investigate the degree of inaccuracy when using *f*_G_ to infer mutant frequencies under fluctuating selection with drift, we performed a computer simulation under 2 different scenarios. Briefly, in a haploid population of *N*_e_ = 1,000, a mutant with an initial frequency of 0.5 was simulated to undergo 2*n* generations of fluctuating selection. In the first scenario, the relative fitness of the mutant was set at 5/6 for the first *n* generations and 6/5 for the next *n* generations, so that *f*_G_ = 1. This scenario may be qualitatively viewed as a 2-generation fluctuating selection if we treat the first *n* generations as one generation with $f_{1}=(5/6{)}^{n}$ and the remaining *n* generations as another generation with $f_{2}=(6/5{)}^{n}$, although the amount of drift in *n* generations is certainly greater than that in one generation. In the second scenario, the relative fitness of the mutant was 5/6 in generations 1, 3, 5, etc. and 6/5 in generations 2, 4, 6, etc. for a total of 2*n* generations, resembling cyclic environmental changes such as days and nights or summers and winters. In both scenarios, consistent with the theoretical prediction that, when *f*_2_ > 1, the expected mutant frequency after 2 generations of fluctuating selection with drift is lower than that predicted under constant selection with *f*_G_, the final mutant frequency was found to be significantly lower than the initial mutant frequency, and this difference enlarges with *n* (blue and green lines in Fig. 3a). The larger bias of mutant frequency in the first scenario than in the second scenario indicates that the frequency of environmental changes also affects the magnitude of the bias. To further validate that the inaccuracy is caused by the nonlinear aspect rather than the linear aspect of the relationship between the current mutant frequency (*q*) and the effect of selection on the allele frequency ($\Delta_{s}$), we specified a hypothetical linear relationship between *q* and $\Delta_{s}$ in the simulation so that $E(q_{1})=fq_{0}$, where $q_{0}$ is the current mutant frequency and $E(q_{1})$ is the expected mutant frequency in the next generation. Indeed, under the hypothetical rule, the expected mutant frequency after the last generation is not significantly different from the initial mutant frequency (red line in Fig. 3a).

In summary, in the presence of drift, using *f*_G_ yields biased mutant frequency inferences. This inference is accurate only in the deterministic case where drift is negligible or when the allele frequency is extremely low or high such that $\Delta_{s}$ varies virtually linearly with *q*.

### A better solution: effective fitness (*f*_E_)

Given the problem with *f*_G_, we propose a more accurate measure of the overall effect of fluctuating selection on a mutant. The idea originates from the constant effect of a given allele frequency change on fixation probability under neutrality. That is, if a neutral allele's frequency is raised from *q* to q+Δq, the probability of fixation of the allele always increases by Δq, regardless of *q*. This insensitivity to the initial allele frequency inspired us to use the mean allele frequency change per generation driven by selection ($\bar{\Delta_{s}}$) as a measure of the per generation effect of selection. Specifically, at any generation *i*, $\Delta_{s,i}=((q_{i-1}f_{i})/(1+q_{i-1}(f_{i}-1)))-q_{i-1}$, where $f_{i}$ is the mutant fitness in generation *i* and $q_{i-1}$ is the mutant frequency at the end of generation *i* − 1. We can then average the allele frequency change caused by selection over generations of interest (from the emergence of the allele to its fixation or loss, or any period of time in between), or $\bar{\Delta_{s}}=(1/n)\sum_{i=1}^{n}\Delta_{s,i}$, where *n* is the number of generations involved. We then define the effective fitness (*f*_E_) of the mutant to be the fitness with which a mutant of frequency 0.5 will have an expected frequency change of $\bar{\Delta_{s}}$, driven by selection, in one generation. That is, $\bar{\Delta_{s}}=(0.5f_{E}/(1+0.5(f_{E}-1)))-0.5$, which means that $f_{E}=(0.5+\bar{\Delta_{s}})/(0.5-\bar{\Delta_{s}})$.

Note that, in addition to the relative fitness of the mutant at each generation, the mutant allele frequency at each generation is also used in calculating $f_{E}$, which should make $f_{E}$ perform better than $f_{G}$. Specifically, in the presence of drift, given *f*_E_ and the number of generations, we expect the same effect of selection on the allele frequency change (i.e. the evolutionary outcome) regardless of the specific temporal variation of fitness, which is not true for *f*_G_. Below, we show that $f_{E}$ provides a virtually unbiased measure of the effect of fluctuating selection in the presence of drift.

Following the logic in proving the inaccuracy of *f*_G_, let us first consider 2 generations. Instead of specifying the relative fitness ($f_{1}$ and $f_{2}$), we now specify the expected allele frequency changes caused by selection in 2 generations ($\Delta_{s,1}$ and $\Delta_{s,2}$). We will investigate whether the expected mutant frequency after 2 generations of fluctuating selection, with or without genetic drift, is equivalent to the mutant frequency resulting from 2 generations of selection with a constant per generation allele frequency change specified by $f_{E}$. The derivation of mutant frequency in the absence of drift is similar to the derivation of the expected mutant frequency in the presence of drift; so only the latter will be presented below.

The number of mutants after the first generation of selection follows $m_{1}∼B(N_{e},q_{0}+\Delta_{s,1})$. Therefore, the mutant frequency after the first generation becomes $q_{1}=m_{1}/N_{e}$, which is a random variable following the scaled binomial distribution with mean $E(q_{1})=q_{0}+\Delta_{s,1}$.

Given *q*_1_, the number of mutants after another generation of selection follows $m_{2}∼B(N_{e},q_{1}+\Delta_{s,2})$. In reality, however, *q*_1_ is a random variable due to the genetic drift in the first generation; consequently, the probability of achieving $\Delta_{s,2}$ varies depending on *q*_1_. Nonetheless, because the mutant frequency change caused by genetic drift is typically tiny in one generation [with the variance equal to $q_{0}(1-q_{0})/N_{e}$], the probability of achieving $\Delta_{s,2}$ is insensitive to the drift-induced variation of *q*_1_. Hence, the expected mutant frequency after the second generation is $E(q_{2})=E(m_{2})/N_{e}=E(q_{1}+\Delta_{s,2})\approx E(q_{1})+\Delta_{s,2}=q_{0}+\Delta_{s,1}+\Delta_{s,2}.$ It is easy to extrapolate the above result to the expected mutant frequency after *n* generations:


$$\begin{array}{c} E(q_{n})\approx q_{0}+\overset{n}{\underset{i=1}{\sum }}\;\Delta_{s,i} \end{array}$$ (6)

Now, let us calculate the expected mutant frequency after *n* generations of selection with a constant per generation allele frequency change specified by *f*_E_. According to the definition of *f*_E_, this constant allele frequency change equals $\bar{\Delta_{s}}=(1/n)\sum_{i=1}^{n}\Delta_{s,i}$. Under the same initial condition and genetic drift, the expected mutant frequency in the final generation can be calculated as follows. The expected mutant frequency after the first generation is $E(q_{1}^{\prime })=q_{0}+\bar{\Delta_{s}}.\;$ The expected mutant frequency after the second generation is $E(q_{2}^{\prime })\approx E(q_{1}^{\prime })+\bar{\Delta_{s}}=q_{0}+\;2\bar{\Delta_{s}}$. So, it is easy to extrapolate this result to the expected mutant frequency after *n* generations:


$$\begin{array}{c} E(q_{n}^{\prime })\approx q_{0}+n\bar{\Delta_{s}}=q_{0}+\overset{n}{\underset{i=1}{\sum }}\;\Delta_{s,i} \end{array}\;$$ (7)

According to (6) and (7), we have $E(q_{n})\approx E(q_{n}^{\prime }).$

It is important to note that in the above derivation, we assumed that $q_{i}+\Delta_{s,i+1}$ and $q_{i}+\bar{\Delta_{s}}$ (for any i∈N) fall within (0,1). This assumption is reasonable as long as the mutant allele is neither fixed nor lost because $\Delta_{s}$ is typically quite small in natural (Waples and Teel 1990; Chen et al. 2019; Rudman et al. 2022) and laboratory (Good et al. 2017; Chen and Zhang 2020; Abdul-Rahman et al. 2021) evolution.

Therefore, we have proved that the expected mutant frequency after an arbitrary number of generations of fluctuating selection approximates that after the same number of generations of selection with a constant per generation allele frequency change corresponding to *f*_E_.

To illustrate the above theoretical finding on *f*_E_, we performed a computer simulation similar to the one used earlier for examining *f*_G_. Briefly, in a haploid population of *N*_e_ = 1,000, a mutant with an initial frequency of 0.5 has a fitness that results in an expected per generation frequency change of $\Delta_{s}$ = 0.045 for *n* generations (see *Materials and Methods* for the specific choice of $\Delta_{s}$). The fitness of the mutant then changed such that $\Delta_{s}$ = −0.045 for another *n* generations. Hence, *f*_E_ = 1 over the 2*n* generations of evolution. Consistent with the theoretical prediction, the final expected mutant frequency under the fluctuating selection with drift is virtually identical to the initial mutant frequency (orange line in Fig. 3a).

### Evolutionary outcomes are better explained by *f*_E_ than *f*_G_

Next, we used computer simulation to evaluate the accuracy and precision of *f*_E_-based and *f*_G_-based inferences of the evolutionary fate of a mutant. Briefly, we considered a mutant with an initial frequency of 0.01 in a haploid population of *N*_e_ = 1,000. The relative fitness of the mutant changed after *t* generations, where *t* follows a Poisson distribution with a mean of 20. The relative fitness switched between $f_{1}=11/10$ and $f_{2}=10/11$, so that *f*_1_*f*_2_  =1. The initial fitness was either $f_{1}$ or $f_{2}$ with equal probabilities. We evolved the population for 200 generations. Due to stochasticity, different simulation runs typically resulted in different values of *f*_E_, *f*_G_, and the final mutant frequency. We collected the first 500 simulation runs with the final mutant frequency of exactly 0.100 and calculated the expected mutant frequency inferred from *f*_E_ or *f*_G_ in each of these runs (see *Materials and Methods*). The distribution of the 500 expected mutant frequencies inferred from *f*_E_ and that inferred from *f*_G_ are shown in Fig. 3b. We computed 2 summary statistics of 500 inferences: (i) bias, which is the difference between the mean inferred value and the true value of 0.100 (Fig. 3b) and (ii) variance, which is the variance of the 500 inferred values. The total inference error is measured by the mean squared difference (MSE) between an inferred value and 0.100, which equals bias^2^ + variance. One can see that, MSE, bias^2^, and variance are all larger for *f*_G_-based inference than for *f*_E_-based inference (Fig. 3c; Supplementary Fig. 3a). We similarly examined simulation runs that resulted in the final mutant frequency of exactly 0.200, 0.300, …, or 0.900 and observed similar trends in MSE, bias^2^, and variance (Fig. 3c; Supplementary Fig. 3a).

Note that our simulation showed that *f*_E_-based inference of the final mutant frequency is not without bias (Fig. 3b). This bias likely originated from an ascertainment bias in the analysis of the simulated data. For example, with an initial frequency of 0.01, a mutant whose frequency drifted more in the positive direction than in the negative direction is less likely to get lost—so is more likely to be collected for analysis. Furthermore, the same mutant is more likely to reach a higher than lower final frequency because of the overall positive effect of drift, which implies that a sampled simulation with a higher final mutant frequency tends to experience a larger positive bias in genetic drift. Due to the ascertainment bias, the mean final mutant frequency observed is slightly higher than that inferred from *f*_E_ and is more prominent for simulations with higher final frequencies (e.g. 0.900).

For both *f*_E_ and *f*_G_, we found that the primary contributor to MSE is the variance of the mutant frequency inference, not bias^2^ (Fig. 3c). When we performed the same simulation with an initial mutant frequency of 0.5 (Supplementary Fig. 2a and b; Supplementary Fig. 3b); the general conclusion remained but the difference between *f*_G_ and *f*_E_ in their performance is smaller, which is mainly owing to the smaller difference in the variance of the inferred mutant frequency. This is probably because the placement of the initial mutant frequency at 0.5 permits a greater effect of selection relative to drift and allows a more balanced distribution of the mutant frequency trajectory. That is, both selection and drift can now occur in positive and negative directions more evenly in a simulation run, such that the positive and negative errors of mutant frequency inferences by *f*_G_ are more easily canceled out along the mutant frequency trajectory.

In addition, we evaluated the relative consistencies of $f_{E}$ and $f_{G}$ with the evolutionary outcome of the mutant (fixed or lost) under fluctuating selection with drift. The same simulations as described above were carried out with an initial mutant frequency of 0.01. At the time of mutant allele fixation or loss, we recorded $f_{E}$, $f_{G}$, and the evolutionary outcome of the mutant (fixation or loss). We collected 5,000 simulation runs with the result of mutant fixation and 5,000 runs with the result of mutant loss, respectively, from which the ROC, or AUC, was calculated for $f_{E}$ and $f_{G}$, respectively. We found that the AUC is larger for $f_{E}$-based inference than for $f_{G}$-based inference of the evolutionary outcome (Fig. 3d). This disparity was less noticeable when we started the simulation with an initial frequency of 0.5 (Supplementary Fig. 2c). However, when we focused on the simulations where $f_{E}$ contradicts $f_{G}$, that is, when (i) $f_{E}>1$ and $\;f_{G}<1$, or (ii) $f_{E}$ < 1 and $f_{G}>1$, we found a clear trend that the mutant is more often fixed than lost under (i) and is more often lost than fixed under (ii) (Supplementary Fig. 2d). Together, these results demonstrate that $f_{E}$ better explains the evolutionary outcome under fluctuating selection with drift than does $f_{G}$.

### Definition of neutrality and the relative effect of selection

With the introduction of $\bar{\Delta_{s}}$ and $f_{E}$, we may evaluate the (effective) neutrality of a mutant subject to fluctuating selection. One simple way is to apply the classic selection coefficient cutoff of $1/(2N_{e})$ for diploids and $1/N_{e}$ for haploids to |*f*_E_ − 1|. However, *f*_E_ is defined under a mutant frequency of 0.5 when the impact of selection on the mutant frequency is maximized. Thus, applying the same selection coefficient cutoff to $f_{E}\;$ and *f*_G_ would have different consequences.

Another way is to define neutrality in a process-specific manner. That is, from the emergence of an allele to its fixation or loss, or any period of time in between, one can calculate the total allele frequency change caused by selection ($\sum_{i=1}^{n}\Delta_{s,i}$) and that caused by drift ($\sum_{i=1}^{n}\Delta_{d,i}$; the subscript “d” stands for drift). We can estimate $\sum_{i=1}^{n}\Delta_{d,i}$ by subtracting $\sum_{i=1}^{n}\Delta_{s,i}$ from the total allele frequency change observed over *n* generations. To quantify the effect of selection on the allele frequency relative to the sum of the absolute effects of selection and drift, we define $C=\frac{\sum_{i=1}^{n}\;\Delta_{s,i}}{\left|\sum_{i=1}^{n}\;\Delta_{s,i}|+|\sum_{i=1}^{n}\;\Delta_{d,i}\right|}$. *C* is positive when the selection increases the allele frequency, and negative, when it reduces the frequency. Note that *C* will be undefined when the denominator is 0, meaning that the relative effect of selection cannot be determined if the frequency change by selection and drift are both 0. We can set an arbitrary maximally allowed value of |C| such as 0.05 to call the overall behavior of the allele effectively neutral. Note that, under this definition, the same $f_{E}$ may be classified as neutral or nonneutral, depending on the actual evolutionary process.

**Fig. 4.**
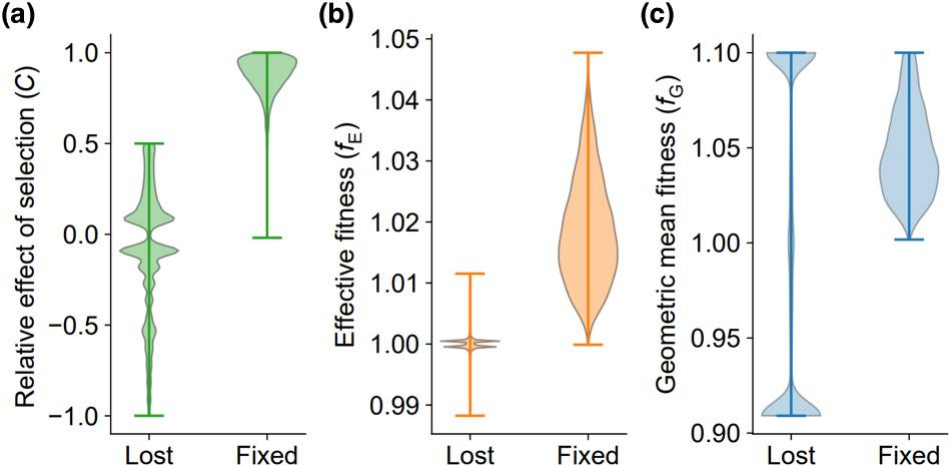
Violin plots showing distributions of *C* (a), *f*_E_ (b), and *f*_G_ (c) at the fixation or loss of a newly arisen allele in simulated evolution. The horizontal bars represent extreme values.

Applying this new metric, we performed simulations to investigate the contribution of (fluctuating) selection to the fixation or loss of a new mutation. All simulation details were the same as described in the preceding section, except that we introduced a single copy of an allele into the population instead of setting the allele frequency at 0.5 to start the simulation. When the allele became fixed or lost, we stopped the simulation and recorded $f_{E}$, $f_{G}$, *C*, and the evolutionary outcome (fixed/lost). We conducted simulations and collected the relevant data from 5,000 runs that resulted in the fixation of the allele and 5,000 runs that resulted in the loss of the allele. We found that the loss of a new mutation was mainly due to drift because over 80% of losses showed |C|<0.5, but only 1.2% were effectively neutral (|C|<0.05) (Fig. 4a). By contrast, most fixations were positively contributed by selection, because almost all fixations had C>0.5 (Fig. 4b). As expected, all fixations were associated with *f*_E_ > 1, and almost all losses were associated with 0.99 < *f*_E_ < 1.01 (Fig. 4b). Interestingly, almost all losses were associated with *f*_G_  *<* 0.95 or >1.05 (Fig. 4c), again demonstrating that *f*_G_ has little explanatory power on the evolutionary outcome of fluctuating selection.

## Discussion

In this study, we showed that the commonly used geometric mean fitness ($f_{G}$) is biased in explaining the outcome of fluctuating selection with drift. We then proposed the effective fitness ($f_{E}$) as a new measure of the overall fitness of an allele under fluctuating selection with drift and demonstrated that $f_{E}$ is more consistent with the evolutionary fate of an allele than $f_{G}$. Our simulations showed that, compared with $f_{G}$-based inference, $f_{E}$-based inference of the final mutant frequency has a much smaller bias and variance, the latter being the major contributor to the inference error. It should be noted that, in practice, $f_{E}$ is not used for predicting allele frequency changes in evolution, because computing $f_{E}$ requires allele frequency information. Rather, the primary merit of this new metric is a more accurate assessment of the contribution of selection to the evolutionary outcome in changing environments.

With a better understanding of *f*_G_ and the invention of *f*_E_, let us revisit the example in Fig. 1 where $f_{G}$ suggests an overall negative selection on the mutant while the mutant frequency trajectory indicates a major contribution of positive selection. In this case, $f_{E}\;$ = 1.002 and the relative selection effect *C* = 0.67, consistent with our intuition from the mutant frequency trajectory. In the present frequency-dependent selection, the fitness and mutant frequency trajectories are such that the effect of negative selection on the mutant frequency is minimized (when the mutant frequency is close to 1), while that of positive selection is maximized (when the mutant frequency is intermediate), diminishing the value of *f*_G_ for inferring the evolutionary outcome.

Our analysis considered only populations with a constant $N_{e}$. However, in the real world, $N_{e}$ often fluctuates widely, which could further increase the effect of genetic drift, making $f_{E}$ even better than $f_{G}$ for evaluating the selective effect in evolution. Note that although the calculation of $f_{E}$ does not require knowing *N*_e_ explicitly, $f_{E}$ can still deal with the genetic drift caused by $N_{e}$ changes. This is because calculating $\;f_{E}$ requires knowing allele frequencies at various generations and allele frequency fluctuations contain the *N*_e_ information.

Effective fitness is also useful in situations where the focal allele at a locus is linked with an allele at another locus that has a fitness effect. In such cases, the allele frequency trajectory is only partially determined by the relative fitness of the focal allele, making it difficult to use the relative fitness of the focal allele alone to assess the contribution of selection to the evolutionary outcome of the focal allele. Because effective fitness captures the allele frequency trajectory information, it can easily overcome the above problem and provide a more robust inference under linked selection.

Our current analysis is limited in that it is based on a discrete-time population genetic model with non-overlapping generations. To be more realistic, future works can explore this topic and extend the concept of effective fitness to continuous-time models of population genetics.

Although $f_{E}$ is useful in theoretical and simulation studies, it currently has a limited empirical utility because its calculation requires the information of allele frequency and relative fitness in every generation, while neither is easy to measure in nature. Here, relative fitness is supposed to be measured independently from the information of allele frequencies by, for example, a separate competition experiment. Compared with the calculation of *f*_E_, the calculation of $f_{G}$ is only slightly easier because it needs the information of relative fitness, but not that of allele frequency. This said, both the allele frequency and relative fitness can be measured in the lab in the context of experimental evolution. As experimental evolution becomes more broadly employed in the mechanistic study of evolution (Garland and Rose 2009), effective fitness may find wider empirical utility in the future.

## Supplementary Material

jkad230_Supplementary_Data

## Data Availability

The authors affirm that all data necessary for confirming the conclusions of the article are present within the article, figures, and Supplementary Material. The computer code used is available at https://github.com/song88180/Effective_fitness. Supplemental material available at G3 online.
