# Supplementary material for: Effective fitness under fluctuating selection with genetic drift
Source: G3 (Bethesda). 2023 Oct 10;13(12):jkad230. doi: 10.1093/g3journal/jkad230 (PMC10700052; doi:10.1093/g3journal/jkad230)
Supplement: jkad230_Supplementary_Data [file jkad230_supplementary_data.zip › G3-2023-404571-TR1_Figure_S1.pdf]

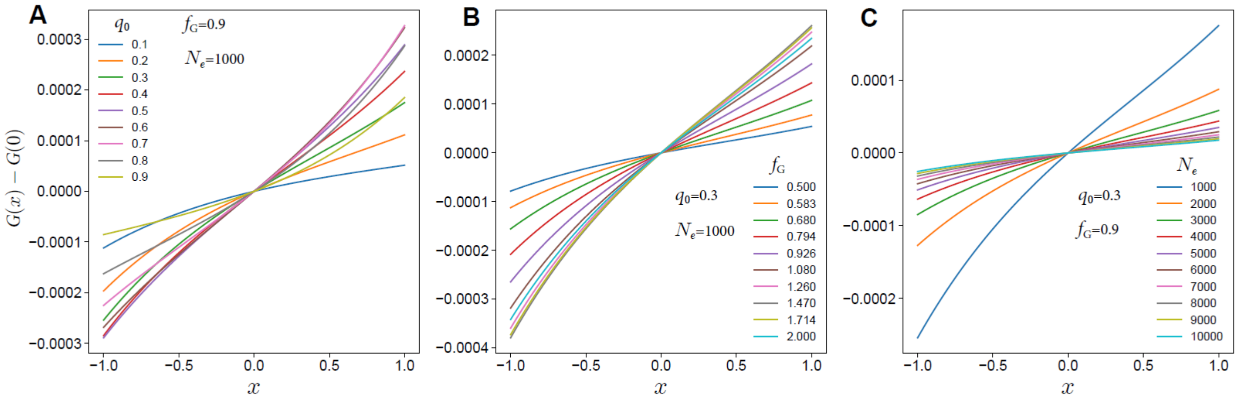

Figure S1. Numerical demonstration that $G(x)$ monotonically increases with x under different q_0 (**A**), f_G (**B**), and N_e (**C**), respectively. Note that $\log_2(f_G)$ is sampled uniformly from -1 to 1 in (B).
