# Supplementary material for: Effective fitness under fluctuating selection with genetic drift
Source: G3 (Bethesda). 2023 Oct 10;13(12):jkad230. doi: 10.1093/g3journal/jkad230 (PMC10700052; doi:10.1093/g3journal/jkad230)
Supplement: jkad230_Supplementary_Data [file jkad230_supplementary_data.zip › G3-2023-404571-TR1_Figure_S2.pdf]

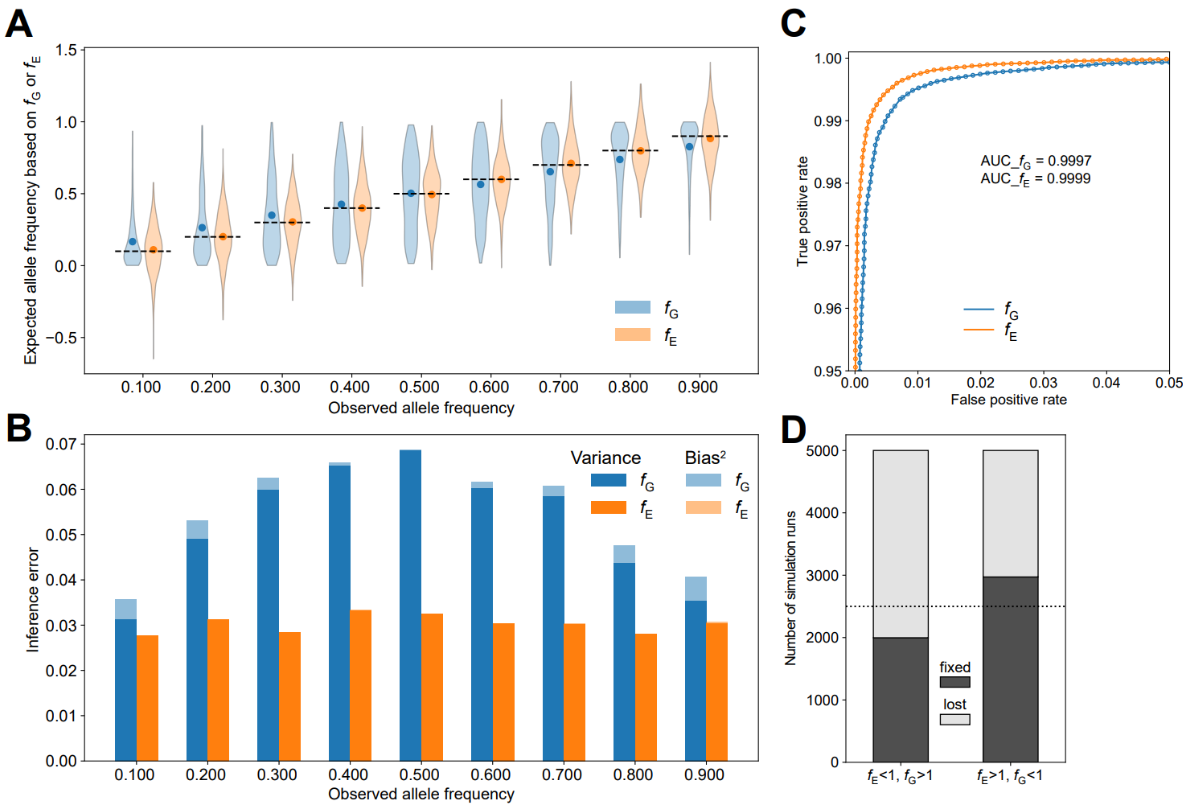

Figure S2. Performance of the effective fitness (f_E) and geometric mean fitness (f_G) in inferring the outcome of simulated evolution with an initial mutant frequency of 0.5. (**A**) Same as **Fig. 3B** except that the initial mutant frequency is 0.5. (**B**) Same as **Fig. 3C** except that the initial mutant frequency is 0.5. (**C**) Same as **Fig. 3D** except that the initial mutant frequency is 0.5. (**D**) Numbers of instances of mutant allele fixation (dark grey) and loss (light grey) from 5000 simulation runs when $f_E < 1$ but $f_G > 1$, or when $f_E > 1$ but $f_G < 1$. The initial mutant frequency is 0.5. The horizontal dotted line represents 2500 instances.
