# Supplementary material for: Effective fitness under fluctuating selection with genetic drift
Source: G3 (Bethesda). 2023 Oct 10;13(12):jkad230. doi: 10.1093/g3journal/jkad230 (PMC10700052; doi:10.1093/g3journal/jkad230)
Supplement: jkad230_Supplementary_Data [file jkad230_supplementary_data.zip › G3-2023-404571-TR1_Figure_S3.pdf]

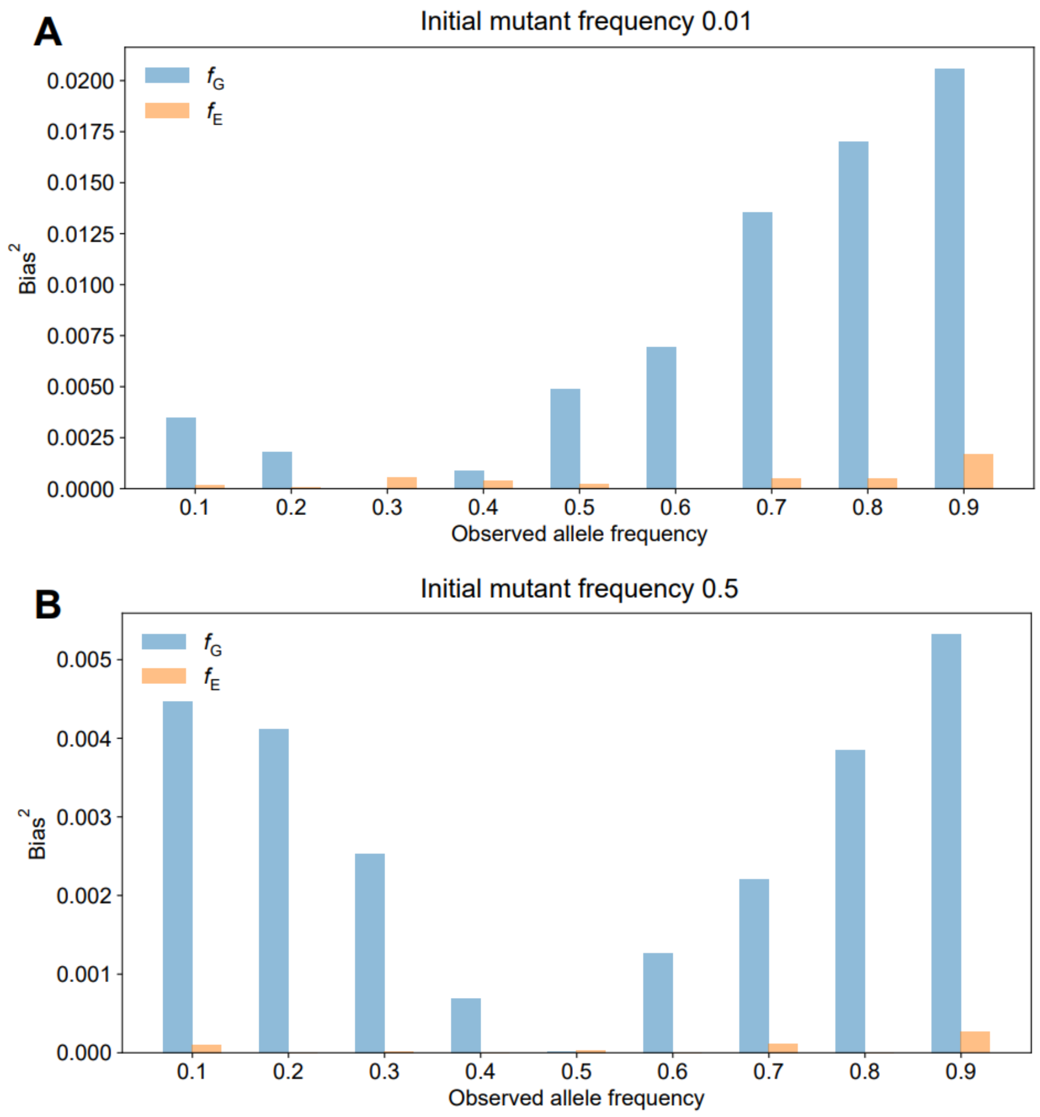

Figure S3. Bias^2 of mutant frequency inference from effective fitness (f_E) and geometric mean fitness (f_G) with initial mutant frequencies of 0.01 (**A**) and 0.5 (**B**). The two panels show in an enlarged fashion the bais^2 presented in Fig. 3C and Fig. S2B, respectively.
